# Procedural Management of Atrial Fibrillation in Older Patients

**DOI:** 10.31083/RCM51902

**Published:** 2026-08-20

**Authors:** Francis Jonathan Ha, Yan Zhao, Hui-Chen Han, Adam J Brown, Nitesh Nerlekar, Emily Kotschet

**Affiliations:** ^1^Victorian Heart Institute and Victorian Heart Hospital, Monash University, Monash Health, Clayton, VIC 3168, Australia

**Keywords:** atrial fibrillation, aged, frail older adults, health services for the aged, anticoagulants, catheter ablation

## Abstract

Atrial fibrillation (AF) is becoming increasingly prevalent worldwide because of population aging and the widespread availability of continuous rhythm monitoring. While medical therapy remains central to management, several procedural strategies are being considered for older patients, including catheter ablation with pulsed field ablation (PFA), atrioventricular node ablation with conduction system pacing (CSP), leadless pacemaker (LPM) implantation, and percutaneous left atrial appendage occlusion (LAAO). Catheter ablation is an established therapy that reduces AF burden and improves quality of life; however, the role of catheter ablation in older patients has historically been limited by higher complication rates and greater arrhythmia recurrence. The emergence of PFA may expand the role of ablation because of its procedural efficiency and favorable safety profile, although randomized data in older patients remain limited. A pace-and-ablate strategy with CSP offers an alternative for older patients with symptomatic persistent AF or tachy-brady syndrome. Indeed, by engaging the native conduction system, CSP provides more physiological ventricular activation and may reduce the risk of pacing-induced cardiomyopathy. LPMs avoid the lead- and pocket-related complications associated with transvenous pacemakers and may be especially advantageous in frail older patients with multiple comorbidities. LAAO is a potential strategy for stroke prevention that is often proposed for patients unable to tolerate long-term anticoagulation, although randomized data remain conflicting. This narrative review evaluates the current evidence supporting these procedures in older patients with AF.

## 1. Introduction

**Fig. 1.**
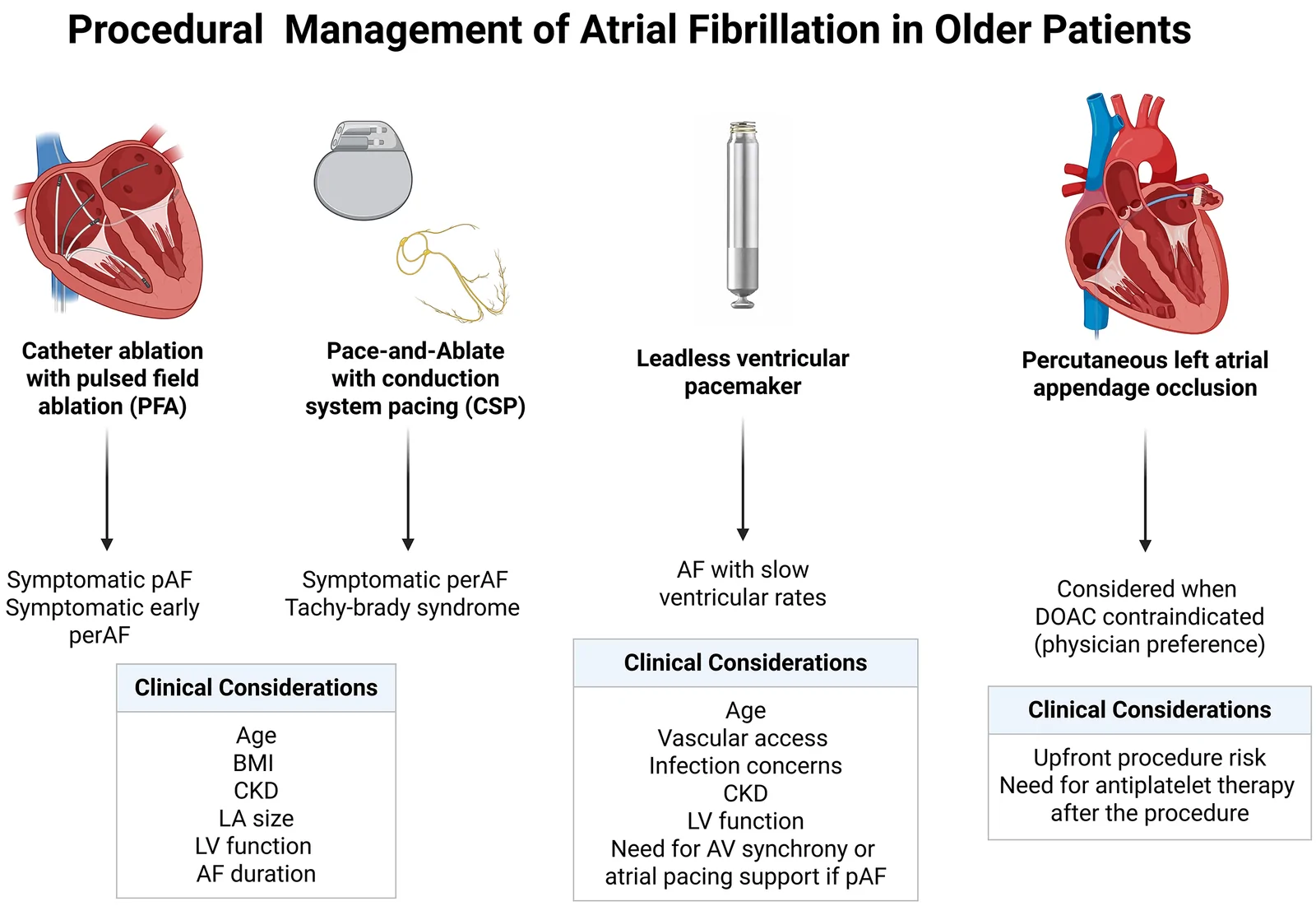
**Potential clinical indications and procedural considerations for older patients with AF**. AF, atrial fibrillation; AV, atrioventricular; BMI, body mass index; CKD, chronic kidney disease; LA, left atrium; LV, left ventricle; DOAC, direct oral anticoagulant; pAF, paroxysmal atrial fibrillation; perAF, persistent atrial fibrillation. Created using BioRender.com.

The global burden of atrial fibrillation (AF) is increasing. An aging population and the availability of continuous rhythm monitoring have led to higher rates of diagnosis and subsequent referral for management [1,2]. In addition to medical therapy, procedural options for AF are continuing to advance. The role of these procedures in older patients must be carefully considered; however, the threshold for offering such procedures to this cohort is decreasing due to increased procedural availability, improved technology, and an expanding body of safety and efficacy data [3]. These procedures include catheter ablation using pulsed field ablation (PFA), a pace-and-ablate strategy with conduction system pacing (CSP), leadless pacemaker (LPM) implantation, and percutaneous left atrial appendage occlusion (LAAO) (Fig. 1). Thus, this narrative review critically evaluates the current evidence base for the various procedures available to older patients with AF. This review recognizes the significant ongoing role of medical therapy in older patients; however, the review specifically aims to clarify the available clinical data on the procedural management of AF in this population.

## 2. Definition of Older Patients

A specific age threshold is not strictly defined for the term “older” patients used in this review. This is because studies vary in the age threshold used to define “older” or “elderly”. Furthermore, a single age cut-off does not readily account for the age distribution within a given country, nor for the comorbidities and frailty of individual patients. In general, most studies included in this review evaluated patients aged 70 years or older. However, as reflected in the literature discussed, many studies have used age thresholds of ≥75 or ≥80 years.

## 3. Catheter Ablation

Catheter ablation is a cornerstone therapy for AF. Sham-controlled randomized trials have shown that catheter ablation significantly reduces AF burden and improves quality of life [4]. Guidelines provide catheter ablation a class I recommendation for symptomatic patients with AF who are intolerant of or have contraindications to antiarrhythmic drugs [5]. Additionally, catheter ablation reduces mortality and heart failure (HF) hospitalizations compared with medical therapy in patients with severe left ventricular (LV) dysfunction, and guidelines similarly provide a class IA recommendation for this specific indication [5,6,7]. However, the applicability of this evidence to older patients has been limited by their historical exclusion from catheter ablation trials. The CABANA trial, which randomized 2204 patients to catheter ablation or medical therapy, found a potential signal toward harm for the primary composite endpoint of death, stroke, serious bleeding, or cardiac arrest among patients aged ≥75 years (308 patients; subgroup analysis) [8]. Numerous systematic reviews and meta-analyses have shown that older patients have higher rates of arrhythmia recurrence and major complications, including stroke, compared with younger patients [9,10]. Thus, it has historically been difficult to justify performing catheter ablation in older patients unless the patient was minimally comorbid, highly symptomatic, or had LV systolic dysfunction.

Nevertheless, the threshold for performing AF ablation in older patients is actively shifting. Data from Danish administrative registries showed a significant increase in the proportion of older patients (≥75 years) undergoing AF ablation, rising from none in 2001 to 9% in 2020 [11]. In that study, age was not an independent predictor of clinical outcomes, including AF-related hospitalizations, repeat ablation, or use of antiarrhythmic drugs (AADs). These data apply to conventional thermal ablation, whether using radiofrequency or cryoballoon technology. However, the advent of PFA could further shift perspectives on ablation in older patients. PFA relies on ultra-short, high-voltage impulses to disrupt the cell membrane [12]. Through its primary mechanism of cell electroporation, PFA is widely considered more tissue-selective for the myocardium while sparing adjacent structures such as the phrenic nerve and esophagus. Hence, many PFA catheters are now commercially available or in development, with new iterations continuing to emerge. The procedural workflow of PFA is significantly more efficient than that of thermal ablation, and several randomized controlled trials (RCTs) have reported non-inferiority in arrhythmia recurrence at 12 months and procedural safety [13,14,15,16]. However, these trials enrolled patients with a median age of only 62–67 years.

Currently, no randomized trial has evaluated PFA exclusively in an older age group. Meanwhile, several multicenter registries have reported outcomes of PFA in older patients (Table 1). A sub-analysis of the EU-PORIA registry, which collected data on AF ablation using the Farapulse PFA system (Boston Scientific, USA) across seven high-volume European centers, reported outcomes for 88 patients aged >80 years [17]. While the overall rate of complications was similar between older and younger patients, the incidence of stroke was higher in the older group (2.3%), including one patient who died following stroke. Notably, at 1-year follow-up, arrhythmia recurrence was similar between groups, with recurrence in one-third of the older patients. Similarly, the prospective global FARADISE registry reported no statistically significant difference in adverse procedural events between 158 patients aged ≥75 years and younger patients; however, the event rate was numerically higher in the older group (2.5% vs. 1.2%, respectively) [18]. Conversely, the ATHENA registry, involving 15 Italian centers, demonstrated a higher rate of arrhythmia recurrence in older patients aged ≥75 years (26.9%) than in younger patients (14.4%; *p* = 0.01) [19]. Overall, there remains at least a numerical signal toward increased complication rates in older patients across these multicenter registries. A large single-center experience also reported an increased risk of major vascular complications (3.1% vs. 0.2%; *p* = 0.003) in older patients (≥75 years) alongside an increased risk of pulmonary edema and transient renal impairment [20].

**Table 1.** **Multicenter registries of pulsed field ablation (PFA) with a pentaspline catheter in older patients**.

Registry (year published)	Study design	Total patients	Older patient cohort	Acute procedural complications	Arrhythmia-free survival
EU-PORIA (2024)	7 European centers	1233	88 (7.1%) >80 years	5.7% vs. 3.5% (*p* = 0.29)	70% vs. 74% (*p* = 0.69) at 12 months
FARADISE (2025)	Global, multicenter	1160	158 (13.6%) ≥75 years	2.5% (≥75 years) vs. 1.2% (<75 years) (*p* = 0.18)	Not reported
ATHENA (2025)	15 Italian centers	1082	108 (10%) ≥75 years	4.6% vs. 2.8% (*p* = 0.14)	73% vs. 86% (*p* = 0.01) at approximately 12 months

The risk of stroke related to PFA is likely multifactorial [21]. Not all catheter designs are equivalent, and several studies have reported an increased risk of silent cerebral emboli with a variable-loop circular catheter compared with other catheter designs [22,23]. Additional considerations include patient comorbidities, including CHA_2_DS_2_-VASc score, interrupted periprocedural anticoagulation, peak activated clotting time in the left atrium, especially during shorter procedures, and the risk of air entrainment during sheath exchange. Vascular access complications must also be considered, especially with the large-profile sheath sizes (up to 16.8 French outer sheath) used by certain PFA systems. An RCT showed that percutaneous suture closure (ProGlide Prostyle, Abbott Cardiovascular, USA) reduced the risk of minor bleeding and shortened time to ambulation after PFA compared with a standard figure-of-eight suture [24].

While data on long-term arrhythmia recurrence outcomes in older patients remain limited, the ADVENT-LTO study, which initially randomized patients to PFA or conventional thermal ablation, showed preserved freedom from arrhythmia intervention at 4 years post-ablation (72.8% with PFA) and fewer repeat ablations with PFA [25]. Again, these data do not apply to older patients, who were excluded from the initial trial, and there remains an unmet need for randomized data in this population.

It is crucial to understand the rationale and objective of catheter ablation in older patients. Given the competing risk of death from multiple non-cardiac causes, a mortality or stroke benefit is unlikely to be demonstrated in this cohort. Instead, the primary aim is to provide symptomatic relief, functional benefit, and improved quality of life through a reduced AF burden [26]. Therefore, patients being considered for ablation should be symptomatic despite antiarrhythmic drug therapy and have a realistic expectation of maintaining sinus rhythm for a reasonable period, accounting for risk factors for recurrence such as cardiovascular comorbidities (*e*.*g*., obesity, hypertension) and left atrial size. Furthermore, the potential benefit of restoring sinus rhythm must outweigh the upfront procedural risk, which requires a transparent discussion with the patient and their family about the level of acceptable risk.

The role of withdrawing therapeutic anticoagulation after successful AF ablation is increasingly being considered. The ALONE-AF trial showed that discontinuation of oral anticoagulation (OAC) after AF ablation reduced the risk of major bleeding with no statistically significant difference in ischemic stroke at 2 years [27]. The OCEAN trial likewise reported no statistically significant difference in the rate of stroke, systemic embolism, or new covert embolic stroke at 3 years after AF ablation between patients receiving rivaroxaban and those receiving aspirin [28]. However, patients aged >80 years and >85 years were excluded from these two trials, respectively, and the mean age of included patients was 63 to 66 years. The median CHA_2_DS_2_-VASc score ranged from 2 to 2.2. Thus, these data cannot be applied to older patients, who are generally more comorbid and have higher CHA_2_DS_2_-VASc scores, especially given their increased likelihood of arrhythmia recurrence compared with younger patients.

Looking ahead, there is growing interest in catheter ablation for older patients. While this trend was already occurring with thermal ablation, this interest may accelerate with PFA. However, caution is warranted given the current lack of randomized data. To date, studies have only evaluated outcomes retrospectively between younger and older patients. While such studies offer some perspective, older patients will inevitably have worse longer-term outcomes because of their comorbidities and inherently increased frailty relative to younger cohorts. Therefore, there is a need to evaluate long-term safety and efficacy in older patients randomized to catheter ablation with PFA versus standard medical therapy.

## 4. Pace and Ablate Strategy

Patients with symptomatic persistent AF or tachy-brady syndrome may benefit from insertion of a permanent pacemaker and atrioventricular node (AVN) ablation, otherwise known as a pace-and-ablate strategy. Beat-to-beat irregularity and the absence of atrial contraction have adverse hemodynamic consequences, including increased filling pressures and LV mechanical stretch, which leads to overall adverse cardiac remodeling [29]. A self-perpetuating cycle may then develop, with further atrial fibrosis and abnormal calcium handling that results in an arrhythmia-induced cardiomyopathy. Regularization of the R–R interval through pacing can often improve cardiac output even in the presence of ongoing AF. However, the pace-and-ablate strategy has generally been viewed as a last-line measure in most patients with AF because of irreversible pacemaker dependence, a consideration that is particularly relevant in younger individuals. Pacemakers and their associated transvenous leads require generator changes and carry a lifetime risk of device- and/or lead-related complications. Furthermore, the potentially detrimental effects of chronic right ventricular (RV) pacing are now well established, as evidenced by the recognition of pacing-induced cardiomyopathy (PICM) [30]. These patients develop iatrogenic ventricular dyssynchrony over time, leading to a decline in LV function and associated HF hospitalizations. PICM is strongly associated with paced QRS duration, and patients with pre-existing LV dysfunction are at higher risk [31].

However, the advent and widespread clinical adoption of CSP could reshape the landscape of the pace-and-ablate strategy for AF. CSP provides the opportunity to capture the native conduction system, resulting in a more physiologic form of pacing. Consequently, ventricular dyssynchrony is substantially reduced compared with conventional RV pacing. This is especially important for patients who are pacemaker-dependent. Initially, CSP was attempted by capturing the His bundle. While this produced a remarkably narrow-paced QRS morphology [32], this pacing approach was limited by procedural challenges in targeting the relatively small anatomical region of the His bundle and by subsequent issues with rising capture thresholds. Owing to these concerns, it was not unusual for operators to implant a backup ventricular lead at the same procedure. There has since been a paradigm shift toward left bundle branch area (LBBA) pacing. The LBBA is a much broader anatomical target, making it easier to access. Accepted pacing outcomes include deep septal, left ventricular septal, and true LBBA capture, whether involving the proximal left bundle branch or fascicular capture, more commonly the inferior/posterior fascicle.

Limited data are currently available on CSP with AVN ablation. Several RCTs have been conducted in patients with atrioventricular block and normal or mildly reduced LV systolic function [33,34,35,36]. The mean age across these trials was 75–77 years, reflecting a more typical older cohort at risk of atrioventricular block. Patients treated with CSP tended to have fewer HF hospitalizations, less PICM, and greater improvement in LV ejection fraction (EF) than those treated with conventional RV pacing. No differences have been observed in all-cause mortality, new-onset AF, or subsequent need for lead revision. Specifically, randomized data are also available for AV node ablation combined with biventricular pacing (CRT). In the APAF-CRT trial, 133 patients with permanent AF and at least one HF hospitalization were randomized to either AVN ablation with CRT or pharmacologic rate control. The trial showed a significant reduction in the primary endpoint of all-cause mortality (hazard ratio (HR), 0.26; 95% confidence interval (CI), 0.10–0.65) with AVN ablation plus CRT after a median follow-up of 29 months. Patients were older (median 72 to 74 years) than those in trials of pulmonary vein isolation (PVI), and 40% had severe LV dysfunction (LVEF ≤35%) [37]. Patients had well-established permanent AF, with 22 to 30 previous electrical cardioversions and had been in AF for 18 months prior. Thus, these findings suggest that older patients with longstanding persistent AF can benefit from a pace-and-ablate strategy with CRT. Similarly, a randomized trial (PACE-FIB trial) is underway to examine this strategy using CSP compared with optimal medical rate control therapy [38]. Importantly, the role of CSP versus PVI catheter ablation in patients with longstanding persistent AF also remains to be clarified, and a randomized trial (Ablate Versus Pace Trial) is underway to examine this question in older patients [39].

Despite promising results from clinical trials of CSP, long-term lead durability data are still needed. Registry data from the LIFE-LBBAP study showed a 0.4% risk of lead tip fracture with stylet-driven leads up to 30 months post-implant [40]. This was higher than the rate seen with lumenless leads (0.04%). Additional concerns have been raised regarding micro-dislodgement, identified during device follow-up as a loss of LBBA capture [41]. The consequences of loss of LBBA capture remain uncertain and may depend on the extent of micro-dislodgement and the resulting type of capture, whether deep septal or standard RV capture.

It remains unclear where the pace-and-ablate strategy with CSP fits within the treatment spectrum for AF. However, it is becoming an increasingly attractive option in older patients with refractory symptomatic atrial tachyarrhythmia despite medical therapy, with the goal of improving quality of life while limiting the risk of PICM and worsening HF. While randomized trials are awaited, the expanding role of PFA may again relegate this strategy to a position behind PVI and non-PV trigger ablation as initial treatment. However, in older patients, it may be reasonable to offer this strategy earlier depending on individual frailty, comorbidities, and AF duration. We await randomized data and long-term clinical follow-up to clarify whether it should play a role earlier in the course of AF management.

## 5. Leadless Pacing

The concept of LPMs originated from efforts to avoid the risks inherent in transvenous pacemakers (TVPMs). Pocket-related complications include infection and hematoma, while transvenous lead-related complications include fracture and dislodgement. Additional risks associated with vascular access for lead placement include pneumothorax and access-site complications in patients with venous thrombosis, prior lymph node clearance, or arteriovenous fistulas. This is particularly relevant in older patients, who are inherently frailer and may have more comorbidities such as chronic obstructive pulmonary disease (COPD), chronic kidney disease requiring renal replacement therapy, and immunosuppression, all of which increase infection risk [42].

Two LPM devices are currently commercially available: Micra (Medtronic, USA) and AVEIR (Abbott, USA). Initial trials (the MICRA IDE trial and LEADLESS II) enrolled older patients (mean age 75 years), with follow-up ranging from 4 to 14 months (Table 2, Ref. [43,44,45]). Implant success rates were high for both devices, at 98–99% [43,44]. Overall complication rates for Micra and AVEIR were 4.0% and 6.7%, respectively. These included cardiac perforation (Micra 1.6%, AVEIR 1.9%), access-site complications (Micra 0.7%, AVEIR 1%) likely associated with the need for large-bore transcatheter femoral access using a 27 French outer sheath, and issues with deployment or dislodgement (Micra 0%, AVEIR 1.0%). An atrial LPM (AVEIR) is also available and can be implanted for sinus node dysfunction or to preserve atrioventricular synchrony, through wireless communication with a ventricular LPM [46]; however, this is typically less relevant in patients who have already developed AF.

**Table 2.** **Clinical trial and post-approval registry data for leadless pacemakers (LPMs)**.

Study		No. of participants	Mean age	Implant success (%)	Mean follow-up in months	Overall complication rate (%)	Pericardial effusion/perforation (%)	Access site complications (%)	Device dislodgement (%)	System revision rate (%)
Clinical trials										
	Micra IDE, 2016 [43]	725	75.9 ± 10.9	99.2	4	4.0	1.6	0.7	0	0.4
	LEADLESS II AVEIR, 2023 [44]	210	75.8 ± 12.1	98	14.4	6.7	1.9	1	1	-
Post-Approval registry										
	Micra PAR, 2024 [45]	1809	75.6 ± 13.4	99.1	51.1	4.1	0.4	0.7	0.2	4.9

Longer-term outcomes have been evaluated in several prospective non-randomized studies and the LPM post-approval registry. In the Micra post-approval registry study of 1809 participants, the mean age at implantation was 75 years, and the major complication rate at five-year follow-up was 4.5%, lower than that observed with TVPM (8.5%) (HR 0.47, 95% CI: 0.36–0.61) [45]. The revision rate at 3 years was 3.2%, predominantly due to CRT device upgrade or increased capture thresholds associated with battery depletion. While this rate was lower than that seen with TVPM (6.6%), the comparison was non-randomized and does not fully account for confounders. Notably, the rate of pericardial effusion or perforation was lower than that reported in clinical trial data (0.44%), which may reflect operator experience and a shift in preference from apical to septal implantation.

When evaluating clinical outcomes in even older patients, leadless pacing has been shown to be successful in those aged ≥85 years, with high procedural success (>98%), no difference in procedure-related complications, and significantly shorter procedure times compared with transvenous pacing [47]. Comparable short-term clinical outcomes have also been shown in nonagenarians (aged ≥90 years) [48]. In a retrospective observational study of older patients (mean age 71–83 years) with high-risk comorbidities, including diabetes, COPD, malignancy, end-stage renal failure, and tricuspid valve disease, LPMs were associated with fewer complications, such as infection, thromboembolism, and dislodgement, as well as fewer reinterventions, compared with TVPM [49]. However, these retrospective datasets are clearly subject to selection bias when identifying suitable candidates for an LPM. Frailty, greater comorbidity burden, susceptibility to infection, and vascular access concerns all influence decision-making. Ongoing issues regarding LPMs include battery longevity, which depends on ventricular pacing burden; the risk of pacemaker syndrome due to loss of atrioventricular synchrony in single-chamber implants; and the risk of PICM due to ventricular dyssynchrony. Nevertheless, LPMs appear to be a viable and safe alternative to transvenous pacing in carefully selected older patients. This is especially relevant in older individuals whose anticipated ventricular pacing needs may still fall within their life expectancy, thereby reducing the likelihood of requiring additional LPM implantation, with or without retrieval of the original device.

## 6. Left Atrial Appendage Occlusion

Anticoagulation remains central to reducing embolic stroke risk in AF. However, clinical judgment and shared decision-making are particularly important in older patients, who often have higher risks of bleeding due to increased frailty, falls, and comorbidity burden. An individual patient-level meta-analysis of four RCTs involving 412 participants with AF and a history of intracranial hemorrhage (ICH) reported that continued OAC reduced major ischemic events without a significant increase in hemorrhagic events or all-cause mortality. However, the low event rates likely limited statistical power to detect these effects [50]. The PRESTIGE-AF trial of 319 participants (mean age 78–79 years) confirmed the benefit of OAC in reducing ischemic stroke, but with a markedly increased risk of ICH [51].

Percutaneous LAAO devices provide a theoretical means of preventing systemic embolism in non-valvular AF and have been considered a potential alternative for patients with contraindications to OAC. The American College of Cardiology/American Heart Association guidelines provide a class IIa recommendation in this context, whereas European guidelines provide a class IIb recommendation [52]. Table 3 (Ref. [53,54,55,56,57]) summarizes findings from RCTs comparing LAAO with OAC. PROTECT-AF was an industry-sponsored RCT of 707 patients with non-valvular AF (mean age 71–73 years, 69% with CHADS_2_-VASc ≥2) randomized to LAAO or warfarin. The investigators reported non-inferiority for the primary efficacy endpoint, a composite of stroke (ischemic and hemorrhagic), systemic embolism, and death [53]. However, there were 22 (4.8%) serious pericardial effusions in the LAAO group. The US Food and Drug Administration requested an additional trial to provide more safety data, given the significant proportion of patients with low stroke risk in the initial PROTECT-AF study. The subsequent PREVAIL trial failed to achieve non-inferiority of LAAO versus warfarin for its overall efficacy endpoint of stroke, systemic embolism, and death ≥7 days post-procedure. However, it had a lower incidence of pericardial effusion (1.9%) [54]. In the era of direct oral anticoagulants (DOACs), the PRAGUE-17 trial compared LAAO with DOAC therapy. In a higher-risk cohort (mean CHA_2_DS_2_-VASc score 4.7 ± 1.5) with a mean age of 73–74 years, the investigators reported non-inferiority of LAAO for a composite endpoint of cardioembolic events, cardiovascular death, major bleeding, and procedure-related complications in 402 patients. However, 10% of procedures failed, and 4.5% of patients experienced a major procedure-related complication [55].

**Table 3.** **Randomized clinical trials (RCTs) comparing left atrial appendage occlusion (LAAO) with oral anticoagulation or physician-directed medical care**.

Trial	Study design	Total patients	Age, years	Stroke risk	Follow-up	Primary endpoint	Results	Procedural complications
PROTECT-AF, 2009 [53]	Non-inferiority: Watchman vs. warfarin	707	71.7–72.7	CHADS_2_ 2.2–2.3	Mean 18 months	Composite of stroke, systemic embolism, or death	8.4% in the LAAO arm and 13.9% in the warfarin arm	Serious pericardial effusion: 4.8%
PREVAIL, 2014 [54]	Non-inferiority: Watchman vs. warfarin	407	74.0–74.9	CHADS_2_ 2.6	Median 12 months	Composite of stroke, systemic embolism, and death	5.3% in the LAAO arm and 2.9% in the warfarin arm	Cardiac perforation or effusion with tamponade 1.9%
PRAGUE-17, 2020 [55]	Non-inferiority: LAAO (Watchman or Amulet) vs. DOAC	402	73.2–73.4	CHA_2_DS_2_-VASc 4.7	Median 19.9 months	Composite of cardioembolic events, cardiovascular death, major bleeding, and procedure-related complications	11.0% in the LAAO arm and 13.4% in the DOAC arm	LAAO-related major complications: 4.5%
CLOSURE-AF (2026) [56]	Non-inferiority: LAAO vs. physician-led medical care	912	77.3–78.5	CHA_2_DS_2_-VASc 5.1–5.2	Median 3 years	Composite of stroke, systemic embolism, major bleeding, or cardiovascular or unexplained death	16.8% in the LAAO arm and 13.3% in the physician-directed medical care arm (per 100 patient-years)	4.0% major bleeding leading to transfusion; 1.1% pericardial tamponade; 4.5% device leak
CHAMPION-AF (2026) [57]	Non-inferiority: Watchman FLX vs. DOAC	3000	71.6–71.8	CHA_2_DS_2_-VASc 3.5	3 years	Composite of cardiovascular death, stroke, or systemic embolism	5.7% in device arm and 4.8% in DOAC arm	Pericardial tamponade: 0.7%; device-related thrombus: 4.8%

DOAC, direct oral anticoagulant; LAAO, left atrial appendage occlusion; FLX, Flexibility.

It is important to remember that this invasive procedure, undertaken with the expectation of reducing future bleeding events, must be weighed against its upfront procedural risks. This is especially relevant in the real-world population most likely to receive LAAO: older, frailer patients with AF and relative or absolute contraindications to therapeutic anticoagulation. Until recently, randomized data in this setting were limited. In addition, such patients inherently have shorter life expectancy, which may reduce the opportunity to realize any long-term stroke benefit and may render the upfront procedural risk disproportionate. The only investigator-initiated trial to examine this clinical dilemma is the CLOSURE-AF trial [56]. This non-inferiority study enrolled patients with AF, an elevated CHA_2_DS_2_-VASc score of ≥2, and either a HAS-BLED score of ≥3 or contraindications to long-term anticoagulation. Patients were randomized to either LAAO or physician-directed medical care, which could include a DOAC if deemed appropriate. Among 912 patients with AF (mean age 77.9 years) at high risk of stroke and bleeding (mean CHA_2_DS_2_-VASc score 5.2, mean HAS-BLED score 3.1) followed for a median of 3 years, the primary endpoint of stroke, systemic embolism, major bleeding, cardiovascular death and unexplained death occurred more often in patients randomized to LAAO than in those receiving medical care (16.8 vs. 13.3 per 100 patient-years). Notably, bleeding might have been expected to be lower in the LAAO group, especially given that 85.1% of the medical therapy arm still received a DOAC; however, it was numerically higher (7.4 vs. 6.2 per 100 patient-years, respectively). Meanwhile, rates of systemic embolism and stroke were also numerically higher in the LAAO group (2.9 vs. 2.8 per 100 patient-years, respectively). Hospitalization for bleeding or cardiovascular events was significantly more frequent with LAAO; 4.5% of patients had residual leak, 4.0% had a major periprocedural bleed, and 1.1% had periprocedural cardiac tamponade. While one could argue that the benefits of LAAO accrue over time, long-term benefit appears unlikely given the average age at enrolment. Currently, this important study cautions against LAAO in frail older patients with elevated CHA_2_DS_2_-VASc scores and perceived relative contraindications to anticoagulation, when compared with physician-directed best medical care.

More recently, the CHAMPION-AF trial was published [57]. This industry-sponsored multicenter trial enrolled patients with AF (mean age 71.7 years, mean CHA_2_DS_2_-VASc score 3.5) randomized to an LAAO device (Watchman FLX) or DOAC therapy. For the primary endpoint of cardiovascular death, stroke, or systemic embolism at 3-year follow-up, event rates were 5.7% in the LAAO arm and 4.8% in the DOAC arm. Stroke rates were numerically higher in the LAAO arm than in the DOAC arm (3.6% vs. 2.5%). For the safety endpoint, procedure- and non-procedure-related bleeding was numerically lower in the LAAO arm (5.9%) than in the DOAC arm (6.4%). Procedure-related pericardial tamponade occurred in 0.7%, and device-related thrombus was observed in 4.8% (63 patients), of whom 37.5% required resumption of DOAC therapy. These data highlight that, in patients suitable for DOAC therapy, LAAO is associated with a numerically higher rate of stroke without a significant reduction in bleeding risk; meanwhile, device-related thrombus is commonly observed and often requires resumption of anticoagulation.

After percutaneous LAA closure, patients in clinical trials still required ongoing OAC plus antiplatelet therapy to allow endothelialization and reduce the risk of device-related thrombus. However, the real-world EWOLUTION registry, which included a large proportion of patients with contraindications to OAC, reported that dual antiplatelet therapy was the predominant post-implant antithrombotic strategy in 60% of patients at 2 years, while 85% were receiving either no antiplatelet therapy or single antiplatelet therapy. The observed stroke rate was 1.3% per year, which was markedly lower than would otherwise be expected in untreated patients [58]. Ongoing randomized trials (ASAP TOO, STROKECLOSE) will further clarify the appropriate post-implant antithrombotic strategy [59].

In summary, LAAO is often considered in selected patients with contraindications to OAC, most commonly due to prior bleeding. The only investigator-initiated RCT in patients at high risk of stroke and bleeding showed inferior outcomes with LAAO compared with physician-directed medical care. Current LAAO practice still requires a period of OAC, antiplatelet therapy, or both to allow endothelialization and reduce the risk of device-related thrombus. Procedure-related complications, operator experience, and shared decision-making are important considerations when evaluating LAAO.

## 7. Role of Artificial Intelligence in AF Management

Artificial intelligence (AI) is becoming nearly ubiquitous and is likely to play an increasing role in the detection and management of AF. This is particularly relevant for older patients, in whom the integration of frailty indices, comorbidities, and electrocardiographic (ECG) findings can be used to screen and identify those at high risk of AF. A cluster-randomized trial involving 30,630 patients aged ≥65 years found that the combination of ECG-based AI and clinical factors could predict incident AF diagnosis at 2-year follow-up, with an area under the receiver operating characteristic curve (AUROC) of 0.78 (95% CI, 0.75–0.82) [60]. A European, multicenter cluster-randomized trial is underway to examine whether mobile health-integrated care combining the Atrial Fibrillation Better Care (ABC) pathway and the multidimensional Comprehensive Geriatric Assessment (CGA) can reduce the 12-month risk of all-cause hospitalizations in patients with AF aged ≥65 years [61]. Applications of AI and machine learning also extend to cardiovascular imaging, in-hospital monitoring, wearable technologies, and electronic health records [62]. Moreover, AI-based algorithms could enhance patient selection for current procedures such as catheter ablation by incorporating patient demographics, pre-existing arrhythmia burden, and bleeding risk. Further studies are expected to clarify this promising area of AF management.

## 8. Conclusion

The role and timing of various procedures in the management of older patients with AF remain variable in clinical practice. Multiple factors must be considered, including patient frailty and comorbidities, access to procedures, operator experience and expertise, and shared decision-making. In the context of PFA, catheter ablation can be considered in older patients with symptomatic AF despite antiarrhythmic drug therapy or in those with LV dysfunction when there is a reasonable likelihood of maintaining sinus rhythm. For older patients with more persistent AF, a pace-and-ablate strategy with CSP can restore heart rate regularity and achieve conduction system capture, thereby mitigating the risk of PICM. Leadless ventricular pacing is an alternative to transvenous pacing in frailer patients with a greater comorbidity burden, with potentially lower risks of infection, dislodgement, and reintervention. Currently, percutaneous LAAO is associated with inferior clinical outcomes (stroke, systemic embolism, or major bleeding) in older patients with high baseline risks of stroke and bleeding, based on limited randomized data. Nevertheless, the therapeutic landscape for AF is expanding and has the potential to improve care for the growing older population affected by this condition.
